# Long-Term Use of Angiotensin Receptor Blockers and the Risk of Cancer

**DOI:** 10.1371/journal.pone.0050893

**Published:** 2012-12-12

**Authors:** Laurent Azoulay, Themistocles L. Assimes, Hui Yin, Dorothee B. Bartels, Ernesto L. Schiffrin, Samy Suissa

**Affiliations:** 1 Centre for Clinical Epidemiology, Lady Davis Institute, Jewish General Hospital, Montreal, Quebec, Canada; 2 Department of Oncology, McGill University, Montreal, Quebec, Canada; 3 Department of Medicine, Stanford University School of Medicine, Stanford, California, United States of America; 4 Department of Global Epidemiology, Boehringer Ingelheim GmbH, Ingelheim, Germany; 5 Department of Medicine, Jewish General Hospital, Montreal, Quebec, Canada; 6 Hypertension and Vascular Research Unit, Lady Davis Institute, McGill University, Montreal, Quebec, Canada; 7 Department of Epidemiology, Biostatistics and Occupational Health, McGill University, Montreal, Quebec, Canada; The Chinese University of Hong Kong, Hong Kong

## Abstract

The association between angiotensin receptor blockers (ARBs) and cancer is controversial with meta-analyses of randomized controlled trials and observational studies reporting conflicting results. Thus, the objective of this study was to determine whether ARBs are associated with an overall increased risk of the four most common cancers, namely, lung, colorectal, breast and prostate cancers, and to explore these effects separately for each cancer type. We conducted a retrospective cohort study using a nested case-control analysis within the United Kingdom (UK) General Practice Research Database. We assembled a cohort of patients prescribed antihypertensive agents between 1995, the year the first ARB (losartan) entered the UK market, and 2008, with follow-up until December 31, 2010. Cases were patients newly-diagnosed with lung, colorectal, breast and prostate cancer during follow-up. We used conditional logistic regression to estimate adjusted rate ratios (RRs) and 95% confidence intervals (CIs) of cancer incidence, comparing ever use of ARBs with ever use of diuretics and/or beta-blockers. The cohort included 1,165,781 patients, during which 41,059 patients were diagnosed with one of the cancers under study (rate 554/100,000 person-years). When compared to diuretics and/or beta-blockers, ever use of ARBs was not associated with an increased rate of cancer overall (RR: 1.00; 95% CI: 0.96–1.03) or with each cancer site separately. The use of angiotensin-converting enzyme inhibitors and calcium channel blockers was associated with an increased rate of lung cancer (RR: 1.13; 95% CI: 1.06–1.20 and RR: 1.19; 95% CI: 1.12–1.27, respectively). This study provides additional evidence that the use of ARBs does not increase the risk of cancer overall or any of the four major cancer sites. Additional research is needed to further investigate a potentially increased risk of lung cancer with angiotensin-converting enzyme inhibitors and calcium channel blockers.

## Introduction

The association between angiotensin receptor blockers (ARBs) and cancer is controversial. A meta-analysis of eight randomized controlled trials (RCTs) published in 2010 found ARBs to be associated with a modest increase in the risk of new cancer diagnoses (relative risk: 1.08; 95% confidence interval (CI): 1.01–1.15) [1]. In contrast, a larger meta-analysis of 70 RCTs published later that year did not find that ARBs or any other antihypertensive agent, when used in monotherapy, was associated with an increased risk of cancer [2]. However, an increased risk of cancer was observed in the subgroup of patients who received a combination of ARBs and angiotensin-converting-enzyme inhibitors (ACEIs) (odds ratio (OR): 1.14; 95% CI: 1.04–1.24), though this effect was driven largely by the ONTARGET trial and was no longer significant when analyzed with a random-effects model [2]. The U.S. Food and Drug Administration also conducted its own meta-analysis of 31 RCTs and found no differences in the rate of cancers in ARB users compared to users of other antihypertensive agents (relative risk: 0.99; 95% CI: 0.92–1.06) [3].

The aforementioned meta-analyses have several methodological limitations. First, they were based on RCTs where cancer was not the primary outcome of interest. Second, they did not consider latency. Lastly, they did not clearly identify whether the excess risk was uniform across all cancers or derived from a subset of sites. The first meta-analysis did report data separately for lung, prostate, and breast cancer, finding a significantly elevated risk only for lung cancer (relative risk: 1.25; 95% CI: 1.05–1.49), but the risks with other cancers were all numerically elevated [1]. Thus, while lung cancer may appear to stand out, the magnitudes of the risks do not rule out a possible association with cancer overall and other cancers in particular.

Four observational studies [4]–[7] have been conducted since the publication of the first meta-analyses [1], [2]. These studies produced conflicting results, ranging from a decreased risk in one study [5], no association in two others [4], [7], and an increased risk in the fourth study [6].

Thus, the primary objective of this large population-based study was to assess whether the use of ARBs is associated with an increased overall risk of lung, colorectal, breast and prostate cancers combined. A secondary objective was to assess whether an increased risk is evident in any of these four most common cancers individually.

## Methods

### Data Source

This study was conducted using the General Practice Research Database (GPRD), a primary care database from the United Kingdom (UK) [8]. The GPRD contains the complete primary care medical record for a representative sample of UK citizens enrolled in more than 650 general practices and numbering over 12 million [9]. The Read classification is used to enter medical diagnoses and procedures, and a coded drug dictionary based on the UK Prescription Pricing Authority Dictionary is used for recording prescriptions. Multiple studies have confirmed the validity of drug exposures and diagnoses in the GPRD [10]–[13].

### Ethics statement

The Independent Scientific Advisory Committee of the GPRD and the Research Ethics Committee of the Jewish General Hospital, Montreal, Canada, approved the study protocol.

### Study population

We conducted a population-based cohort study using a nested case-control analysis. The cohort consisted of all patients who prescribed an antihypertensive agent between January 1, 1995, the year the first ARB (losartan) entered the UK market, and December 31, 2008, with follow-up until December 31, 2010. Cohort entry was defined as the date of a first prescription for an antihypertensive agent. Patients were required to have at least two years of up-to-standard medical history in the GPRD prior to the first prescription for an antihypertensive agent. Patients with less than two years of medical history in the GPRD had their cohort entry moved forward in time to the first antihypertensive occurring on or after their second anniversary of registration in the GPRD. Finally, patients with a history of the cancers under study (lung, colorectal, breast and prostate) at any time prior to cohort entry were excluded. Thus, all patients in the cohort were followed until a first ever diagnosis of one of the four cancers of interest (lung, colorectal, breast, and prostate), death from any cause, end of registration with the general practice, or end of the study period (December 31, 2010), whichever came first.

### Case-control selection

Nested case-control analyses were performed to investigate the association between the use of ARBs and the risk of the four most common cancers (lung, colorectal, breast, and prostate), identified on the basis of Read codes. The date of each case's cancer diagnosis was defined as their index date. Up to 10 controls were randomly selected and matched to each case on age (year of birth), sex (when applicable), calendar year of cohort entry, prevalent user status (defined by any prescription for an antihypertensive agent during the two-year period prior to cohort entry), and duration of follow-up. Cancer diagnoses, including lung, colorectal, breast, and prostate cancer, have shown high validity in the GPRD, with sensitivities and positive predictive values exceeding 90% [14]–[17] and with case ascertainment rates comparable to UK cancer registries [18].

### Exposure assessment

For cases and controls, we obtained all information on antihypertensive prescriptions between cohort entry and index date. These consisted of diuretics, beta-blockers, ACEIs, ARBs, calcium channel blockers (CCBs), and other antihypertensives (alpha-blockers, vasodilators, centrally-acting antihypertensive, ganglion-blocking drugs, renin inhibitors).

Patients were grouped into one of the following five mutually exclusive exposure groups defined hierarchically by *ever* use of 1) ARBs, 2) ACEIs, 3) CCBs, 4) other antihypertensives (such as alpha-blockers), and 5) diuretics and/or beta-blockers. The latter group served as the reference category for all analyses since they have not been previously associated with an increased risk of cancer, unlike CCBs and ACEIs [14], [19]. For all of the above, exposures initiated in the year immediately prior to index date were excluded in order to account for a biologically meaningful latency time window, as it is unlikely that a drug will induce a cancer after such a short exposure duration.

### Potential confounders

The models were adjusted for co-morbid clinical conditions and exposures (measured at any time from at least 2 years prior to cohort entry up to one year before the index date) known to be associated with the cancers under study that might also influence the choice of antihypertensive therapy: excessive alcohol use, smoking status, body mass index, hypertension, congestive heart failure, coronary heart disease, diabetes, previous cancer (other than non-melanoma skin cancer and those under study), and the ever use of aspirin, other non-steroidal anti-inflammatory drugs (NSAIDs), and statins. For the cancer site specific analyses, models were further adjusted for cholecystectomy, inflammatory bowel disease (consisting of Crohn's disease and ulcerative colitis), and a history of polyps for colorectal cancer; oophorectomy, use of hormone replacement therapy, and prior use of oral contraceptives for breast cancer; and benign prostatic hyperplasia, prostatitis, and use of 5-alpha reductase inhibitors (finasteride or dutasteride) for prostate cancer.

### Statistical analysis

For all cancers combined and for each cancer, we used multivariate conditional logistic regression adjusted for the confounders listed above to estimate RRs of cancer incidence associated with ARB use as well as 95% CIs. Our primary analyses consisted of estimating RRs for all four cancers combined and for each cancer site separately associated with *ever* use of ARBs between cohort entry and the year prior to index date compared to the use during this same time period of diuretics and/or beta-blockers.

We also conducted four additional secondary analyses among patients deemed to be ever users of ARBs in the primary analysis. In the first analysis, we assessed whether the risk of cancer overall varied according to *time since initiation* of an ARB. This variable was calculated as the time span between a first ARB prescription and index date. In the second analysis, *cumulative duration of use* was calculated by summing the durations of all ARB prescriptions until index date. In the third analysis, we assessed whether the risk varied as a function of the *cumulative dose* of ARB received during the time between cohort entry and index date. *Cumulative dose* was defined daily doses (DDDs) to standardize the varying doses and potencies of the different ARBs prescribed. All three of the aforementioned dose-response variables were entered as tertiles in the models based on the distribution of use in the controls. Finally, in the fourth analysis, we assessed whether concurrent use of ARBs with ACEIs (defined as the prescription coverage of these drugs overlapping each for at least one day) was associated with an increased risk of cancer overall, and for each cancer separately.

We conducted five sensitivity analyses to assess the robustness of the findings. First, we repeated the primary analysis by varying the length of the latency time window to two years. Second, the cohort included both new and prevalent users of antihypertensive drugs, and thus we stratified cases and matched controls on their prevalent user status (which was a matching factor) to determine the risk of cancer overall varied between these two groups. Third, we assessed potential misclassification of exposure by redefining ever use of the different exposure groups as receiving at least four prescriptions within a 12-month period, thus minimizing the inclusion of patients with irregular or sporadic use of these drugs. Fourth, to assess the impact of including patients with other cancers, we repeated the analyses by excluding cases and matched controls that were previously diagnosed with cancers other than the ones under study.

Finally, as an alternate to the hierarchical exposure definition described above, we repeated the primary analysis by entering the exposure groups as non-mutually exclusive variables in the model, thereby estimating their independent effects. All analyses were conducted with SAS version 9.2 (SAS Institute, Cary, NC).

## Results

**Figure 1:**
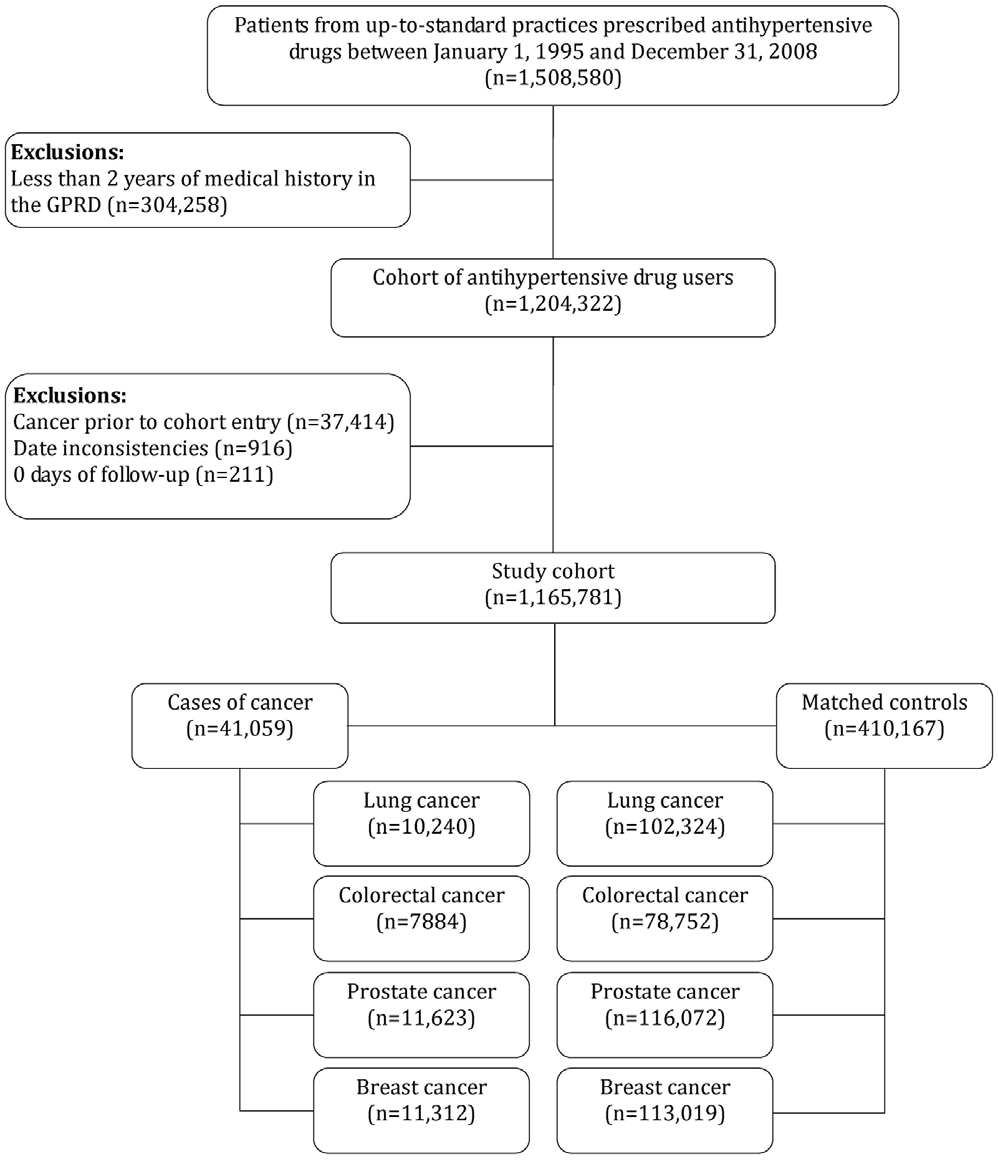
Study flow chart.

A total of 1,165,781 patients met the inclusion criteria (Figure 1). The mean (standard deviation [SD]) age at cohort entry was 63.4 (14.6) years, 525,195 (45.0%) were males, and the mean (SD) duration of follow-up was 6.4 (3.9) years. At cohort entry, 48,830 (4.2%) were prescribed an ARB, 573,171 (49.2%) diuretics and/or beta-blockers, 299,901 (25.7%) ACEIs, 213,124 (18.3%) CCBs, while 30,755 (2.6%) were prescribed other antihypertensive agents.

During the 7,417,010 person-years of follow-up, 41,059 patients were diagnosed with one of the cancers under study (overall rate of 554/100,000 (95% CI: 548–559) persons per year). Table 1 presents the characteristics of the cases and the 410,167 matched controls. At index date, compared to controls, cases were more likely to have used alcohol excessively, to have smoked, and to have a history of cancer (other than non-melanoma skin cancer). Overall, users of ARBs and ACEIs were more likely to have been obese, to have ever smoked and have diabetes, while being more likely to have used aspirin, statins, and NSAIDs than users of other antihypertensive drugs (see Tables S1, S2, S3, and S4).

**Table 1:** Characteristics of cases and matched controls at index date.

	Cases	Controls
Number	41,059	410,167
Age (years), mean (SD)*	72.4 (10.2)	72.4 (10.1)
Duration of follow-up (years), mean (SD)*	5.5 (3.3)	5.5 (3.3)
New AHD users, n (%)*	18,856 (45.9)	188,338 (45.9)
Males, n (%)*	21,586 (52.7)	215,585 (52.7)
Excessive alcohol use, n (%)	3653 (8.9)	30,476 (7.4)
Body mass index, n (%)		
<18.5	539 (1.3)	4312 (1.0)
18.5–25	8078 (19.7)	77,956 (19.0)
25–30	10,189 (24.8)	104,553 (25.5)
≥30	6757 (16.5)	70,319 (17.1)
Unknown	15,496 (37.7)	153,027 (37.3)
Smoking status, n (%)		
Ever	23,287 (56.7)	200,366 (48.8)
Never	15,661 (38.1)	187,034 (45.6)
Unknown	2111 (5.1)	22,767 (5.5)
Hypertension, n (%)	28,778 (70.1)	289,536 (70.6)
Congestive heart failure, n (%)	3229 (7.9)	29,720 (7.3)
Coronary heart disease, n (%)	4161 (10.1)	42,090 (10.3)
Previous cancer, n (%)	4784 (11.6)	37,435 (9.1)
Diabetes, n (%)	6351 (15.5)	64,193 (15.6)
Aspirin, n (%)	20,383 (49.6)	201,063 (49.0)
Statins, n (%)	16,730 (40.7)	166,629 (40.6)
NSAIDs, n (%)	22,712 (55.3)	224,182 (54.7)
Diuretics and/or beta blockers, n (%)‡	34,808 (84.8)	347,776 (84.8)
ARBs, n (%)‡	5583 (13.6)	56,817 (13.9)
ACEI, n (%)‡	19,910 (48.5)	199,737 (48.7)
CCB, n (%)‡	20,285 (49.4)	198,892 (48.5)
Other antihypertensive, n (%)‡	1654 (4.0)	16,844 (4.1)

Abbreviations: SD, standard deviation; AHD, antihypertensive drug; NSAID, non-steroidal anti-inflammatory drug.

* Matching variables (along with year of cohort entry).

‡ Non-mutually exclusive categories.

**Figure 2:**
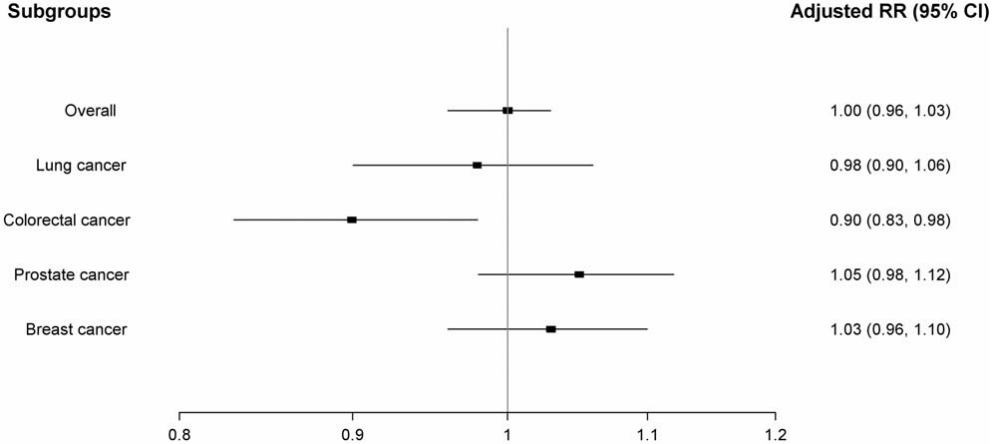
Adjusted rate ratios of specific cancers associated with use of angiotensin receptor blockers relative to the use of diuretics or beta-blockers.

Table 2 and Figure 2 present the results of our primary analyses. When compared to diuretics and/or beta-blockers, ever use of ARBs was not associated with an increased risk of cancer overall (adjusted RR: 1.00; 95% CI: 0.96–1.03). When the use of ARBs was further categorized according to *time since initiation*, *cumulative duration* and *cumulative dose*, no dose-response was observed, with all point estimates around the null value.

**Table 2:** Crude and adjusted rate ratios of cancer associated with antihypertensive agents relative to diuretic and/or beta-blocker use.

	Cases/Controls*	Crude RR	Adjusted RR (95% CI)†
Diuretics and/or beta-blockers	10,091/101,723	1.00	1.00 (Reference)
ARBs	5583/56,817	0.99	1.00 (0.96–1.03)
ARBs with ACEIs‡	2422/24,431	1.00	1.00 (0.95–1.05)
ARBs without ACEIs	3161/32,386	0.99	0.99 (0.95–1.03)
ACEIs	16,035/160,396	1.01	1.00 (0.97–1.03)
CCBs	8622/83,973	1.04	1.02 (0.99–1.05)
Other antihypertensives	728/7258	1.01	0.99 (0.91–1.08)
**ARBs: Time since initiation (years)** §			
≤2.95	1851/18,768	1.00	1.00 (0.95–1.05)
2.96–5.28	1933/19,311	1.01	1.01 (0.96–1.07)
>5.28	1799/18,738	0.97	0.97 (0.92–1.03)
**ARBs: Cumulative duration (years)** §			
≤1.53	1844/18,754	0.99	0.99 (0.94–1.05)
1.54–3.48	1914/19,322	1.00	1.00 (0.95–1.06)
>3.48	1825/18,741	0.98	0.98 (0.93–1.04)
**ARBs: Cumulative dose (DDDs)** §			
≤392	1410/14,383	0.99	0.99 (0.93–1.05)
393–1456	2106/21,307	1.00	1.01 (0.96–1.06)
>1456	2067/21,127	0.99	0.99 (0.94–1.05)

Abbreviations: RR, rate ratio; CI, confidence interval; ARB, angiotensin receptor blocker; ACEI, angiotensin-converting enzyme inhibitor; CCB, calcium channel blocker; DDD, defined daily doses.

* Cases and controls were matched on year of birth, year of cohort entry, sex, prevalent user status, and duration of follow-up.

† Adjusted for excessive alcohol use, body mass index, smoking, diabetes, previous cancer, and ever of aspirin, statins, and NSAIDs.

‡ Defined as receiving prescriptions for both agents on the same day on at least one occasion.

§ Dose-response analyses conducted among the 5583 cases and 56,817 controls exposed to ARBs. Categories based on tertiles.

Table 3 presents the results of the secondary objective. Ever use of ARBs was not associated with risk of lung, prostate, and breast cancer, with the RRs ranging from 1.00 to 1.02. In contrast, the use of ARBs was associated with a modest decreased risk of colorectal cancer (adjusted RR: 0.88; 95% CI: 0.81–0.96). This decreased risk in colorectal cancer was not specific to ARBs, as it was also observed among users of other antihypertensive drugs with adjusted RRs ranging from 0.87 to 0.90. We conducted a post-hoc analysis where all exposure groups were compared to beta-blockers users only as prior studies have suggested an increased risk of colorectal cancer among users of diuretics [20]. This analysis revealed that compared to beta-blockers, none of the antihypertensive drugs were associated with a decreased risk of colorectal cancer (ARBs: adjusted RR: 0.94; 95% CI: 0.83–1.06; ACEIs: adjusted RR: 0.93; 95% CI: 0.83–1.03; CCBs: adjusted RR: 0.96; 95% CI: 0.86–1.07; diuretics: adjusted RR: 1.08; 0.97–1.21; other antihypertensives: adjusted RR: 0.87; 95% CI: 0.64–1.18).

**Table 3:** Crude and adjusted rate ratios of lung, colorectal, prostate and breast cancer associated with antihypertensive agents relative to diuretic and/or beta-blocker use.

	Cases/Controls*	Crude RR	Adjusted RR (95% CI)†
**Lung cancer**	**10,240/102,324**		
Diuretics and/or beta-blockers	2153/24,426	1.00	1.00 (Reference)
ARBs	1258/14,487	1.00	1.01 (0.93–1.09)
ARBs with ACEIs‡	559/6265	1.02	1.01 (0.91–1.12)
ARBs without ACEIs	699/8222	0.97	1.00 (0.91–1.09)
ACEIs	4200/39,668	1.21	1.13 (1.06–1.20)
CCBs	2374/21,189	1.29	1.19 (1.12–1.27)
Other antihypertensives	255/2554	1.13	1.05 (0.91–1.21)
**Colorectal cancer**	**7884/78,752**		
Diuretics and/or beta-blockers	1991/18,730	1.00	1.00 (Reference)
ARBs	1106/11,148	0.93	0.88 (0.81–0.96)
ARBs with ACEIs‡	474/4812	0.92	0.87 (0.78–0.97)
ARBs without ACEIs	632/6336	0.93	0.89 (0.81–0.98)
ACEIs	3001/30,466	0.92	0.87 (0.81–0.93)
CCBs	1594/16,382	0.91	0.90 (0.84–0.97)
Other antihypertensives	192/2026	0.89	0.89 (0.76–1.04)
**Prostate cancer**	**11,623/116,072**		
Diuretics and/or beta-blockers	2486/24,324	1.00	1.00 (Reference)
ARBs	1553/15,182	1.00	1.01 (0.94–1.08)
ARBs with ACEIs‡	668/6630	0.84	0.98 (0.90–1.08)
ARBs without ACEIs	885/8552	1.01	1.01 (0.93–1.10)
ACEIs	4849/50,211	0.94	0.94 (0.90–0.99)
CCBs	2573/24,793	1.02	1.02 (0.96–1.08)
Other antihypertensives	162/1562	1.02	1.02 (0.86–1.20)
**Breast cancer**	**11,312/113,019**		
Diuretics and/or beta-blockers	3461/34,243	1.00	1.00 (Reference)
ARBs	1666/16,000	1.03	1.02 (0.95–1.09)
ARBs with ACEIs‡	721/6724	1.06	1.04 (0.95–1.14)
ARBs without ACEIs	945/9276	1.01	1.00 (0.92–1.08)
ACEIs	3507/35,521	0.98	0.97 (0.92–1.02)
CCBs	2070/21,160	0.97	0.98 (0.92–1.04)
Other antihypertensives	608/6095	0.99	0.97 (0.89–1.07)

Abbreviations: RR, rate ratio; CI, confidence interval; ARB, angiotensin receptor blocker; ACEI, angiotensin-converting enzyme inhibitor; CCB, calcium channel blocker.

* Cases and controls were matched on year of birth, year of cohort entry, sex, prevalent user status, and duration of follow-up.

† All models were adjusted for excessive alcohol use, body mass index, smoking, diabetes, previous cancer, and ever of aspirin, statins, and NSAIDs. In addition, cholecystectomy, inflammatory bowel disease and history of polyps for colorectal cancer; benign prostatic hyperplasia, 5-alpha reductase inhibitors, and number of PSA tests for prostate cancer; oophorectomy, use of hormone replacement therapy, and prior use of oral contraceptives for breast cancer.

‡ Defined prescriptions of both agents overlapping each other for at least one day.

**Figure 3:**
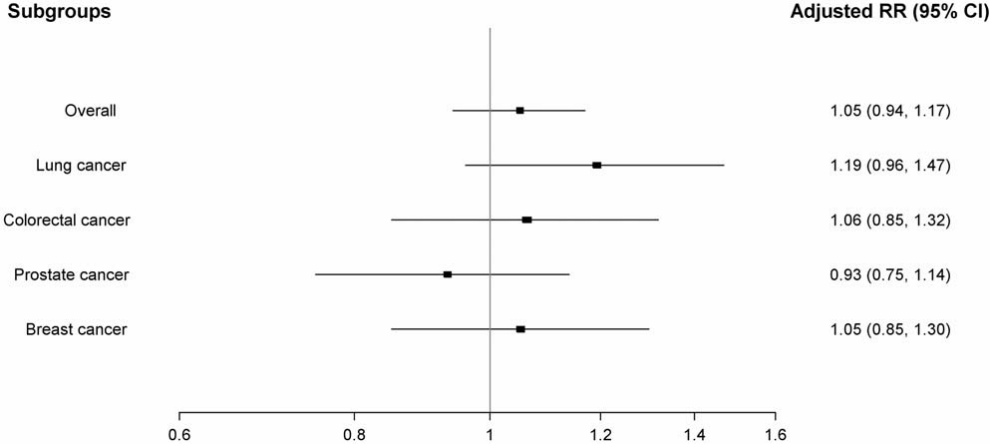
Adjusted rate ratios of specific cancers associated with use of angiotensin receptor blockers in combination with angiotensin-converting enzyme inhibitors relative to the use of diuretics or beta-blockers.

With respect to lung cancer, the use of ACEIs and CCBs were associated with modest increased risks (13% and 19%, respectively). These increased risks were not observed for the other cancer sites. Finally, the concurrent use of ARBs with ACEIs was not associated with an increased risk of cancer overall (Table 2 and Figure 2) or with any of the four specific cancer sites (Table 3 and Figure 3).

### Sensitivity analyses

In the first sensitivity analysis, we varied the latency time window from one year to two years. This analysis produced results consistent with those of the primary analysis (see Table S5). In the second analysis, similar results were observed after stratifying cases and matched controls on prevalent user status (see Table S6). In the third analysis, redefining exposure as receiving at least four prescriptions within a 12-month period led to nearly identical results as that of the primary analysis, indicating that exposure misclassification was likely minimal (ARBs: adjusted RR: 1.00; 95% CI: 0.96–1.04; ACEIs: adjusted RR: 1.00; 95% CI: 0.96–1.04; CCBs: adjusted RR: 1.03; 95% CI: 1.00–1.07; other antihypertensives: adjusted RR: 1.00; 95% CI: 0.96–1.04). In the fourth, excluding patients with any cancer prior to cohort entry led to nearly identical results of that of the primary analysis (see Table S7). Finally, entering the exposure groups as non-mutually exclusive groups in the models did not materially change the results (ARBs: adjusted RR: 0.99; 95% CI: 0.96–1.02; ACEIs: adjusted RR: 0.99; 95% CI: 0.97–1.01; CCBs: adjusted RR: 1.04; 95% CI: 1.01–1.06; other antihypertensives: adjusted RR: 0.98; 95% CI: 0.93–1.03; diuretics and/or beta-blockers: adjusted RR: 1.00; 95% CI: 0.97–1.03) (see Table S8).

## Discussion

The results of this large population-based study involving close to 1.2 million patients treated with antihypertensive agents do not support the hypothesis that the use of ARBs, when compared to diuretics and/or beta-blockers, is associated with an increased risk of cancer overall or with any of the four most common cancers. In fact, the risk of colorectal cancer was modestly decreased. On the other hand, ACEIs and CCBs were associated with a modest increase in the risk of lung cancer.

Four observational studies have been conducted [4]–[7] since the publication of the initial meta-analyses of clinical trials [1], [2]. In two cohort studies, the use ARBs was not associated with an increased risk of cancer overall, when compared to ACEIs (adjusted RR: 0.99; 95% CI: 0.95–1.03 and adjusted HR: 1.03; 95% CI: 0.99–1.06, respectively) [4], [7]. Our results are consistent with these studies although we used a different reference group consisting of patients exposed to diuretics and/or beta blockers. Our large numbers also allowed us to exclude a 4% increased risk based on the upper limit of our 95% confidence intervals. We note that this upper limit is lower than the 8% increased risk found in the first meta-analysis [1]. However, our results differ greatly from the two other observational studies [5], [6]. Specifically, in one cohort study [5], the use of ARBs was associated with a 34% decreased risk of cancer when compared to non-use (hazard ratio: 0.66; 95% CI: 0.63–0.68) but this study likely suffered from substantial immortal-time bias [21]. Finally, in another case-control study [6], all antihypertensive drugs were statistically associated with an increased risk of cancer, with ORs ranging from 1.12 for ARBs to 1.37 for thiazide diuretics [6] but no dose-response relationship between exposure and outcome could be established. As acknowledged by the authors [6], these results were likely affected by confounding by indication, since antihypertensive users were compared to patients from the general population, and thus differences in disease status and cancer screening may have explained the findings.

While our study provides additional evidence in support of the hypothesis that the use of ARBs does not promote any of the four most common cancers, ACEIs and CCBs may be associated with a 13% and 19% increased risk of lung cancer. Although this analysis was part of our secondary objective and should be considered exploratory, it appears to corroborate previous findings that ACEIs in particular may be associated with an increased risk of lung cancer [4], [7]. In one study, when compared to ACEIs, the use ARBs was associated with a 14% to 20% decreased risk of certain lung cancer subtypes (small-cell carcinoma, squamous cell, and others/unspecified types) [4]. These decreased risks may possibly be related to the purported anti-tumor effects of certain ARBs [22], or to a carcinogenic effect of ACEIs. It is also conceivable that detection bias may have led to an overestimation of lung cancers in users of ACEIs through the performance of more frequent chest x-rays to investigate an ACEI induced cough. Unfortunately, chest x-rays are not well recorded in the GPRD, and thus it was not possible to adjust for this variable in the models. As for the increased risk observed with CCBs, there is currently no clear mechanism through which these drugs may increase the risk of this cancer, although such an association has been previously reported [23]. Finally, a decreased risk of colorectal cancer was observed with all antihypertensive drugs (ARBs, ACEIs, CCBs, and other antihypertensive drugs) when compared to diuretics and/or beta-blockers. However, in a post-hoc analysis, this association was no longer present when the reference group was restricted to beta-blockers only, with all point estimates closer to the null value. Thus, it appears that diuretics may be associated with a modest increased risk of colorectal cancer incidence, as previously reported by others [20].

This population-based study has a number of strengths and some limitations. First, we assembled a large population-based cohort close to 1.2 million patients treated with antihypertensive drugs, followed for up to 16 years enabling the identification of a substantial number of cancer cases. Second, we eliminated the possibility of recall bias by using the pre-recorded exposure histories in the GRPD. Third, drug information in the GPRD represents prescriptions written by general practitioners with no information on patient compliance. However, such non-differential misclassification of exposure would have biased the results towards the null. Fourth, a limitation of the GPRD is the lack of information on certain cancer risk factors such as occupational exposures, race/ethnicity, and family history. We believe it unlikely that the internal validity of this study was threatened by a differential distribution between exposure groups. Fifth, residual confounding due to unmeasured or incompletely measured covariates such as the severity of hypertension remains a concern, although prior studies suggest a complex relationship between hypertension and cancer [24], [25]. Finally, a strength of the GPRD database is that it contains information on a number of important confounders, such as BMI, excessive alcohol use, and smoking which is usually not accessible in other administrative databases.

In summary, the results of this large population-based study of patients treated with antihypertensive agents and followed for an average of 6.4 years, provides further evidence that the use of ARBs does not increase the risk of any of the four most common cancers, namely lung, breast, prostate, and colorectal. However, an increased risk of lung cancer was observed with ACEIs and CCBs, although this was a secondary analysis, and thus additional studies are needed to investigate this specific question. Together with the evidence from other large observational studies, this study should reassure treating physicians and patients about the carcinogenic potential of ARBs.

## Supporting Information

Table S1 **Characteristics of antihypertensive exposure groups among controls for lung cancer at index date.**
(DOC)

Table S2 **Characteristics of antihypertensive exposure groups among controls for colorectal cancer at index date.**
(DOC)

Table S3 **Characteristics of antihypertensive exposure groups among controls for prostate cancer at index date.**
(DOC)

Table S4 **Characteristics of antihypertensive exposure groups among controls for breast cancer at index date.**
(DOC)

Table S5 **Crude and adjusted rate ratios of cancer associated with antihypertensive agents relative to diuretic and/or beta-blocker use (two years of latency time window).**
(DOC)

Table S6 **Crude and adjusted rate ratios of cancer associated with antihypertensive agents relative to diuretic or beta-blocker use, stratified by new/prevalent user status.**
(DOC)

Table S7 **Crude and adjusted rate ratios of cancer associated with antihypertensive agents relative to diuretic or beta-blocker use, excluding any cancer before cohort entry.**
(DOC)

Table S8 **Crude and adjusted rate ratios of cancer associated with antihypertensive agents relative to diuretic and/or beta-blocker use, (non-mutually exclusive exposure groups).**
(DOC)

## References

[1] Sipahi I, Debanne SM, Rowland DY, Simon DI, Fang JC (2010) Angiotensin-receptor blockade and risk of cancer: meta-analysis of randomised controlled trials. Lancet Oncol 11: 627–636. 20542468 10.1016/S1470-2045(10)70106-6 PMC4070221

[2] Bangalore S, Kumar S, Kjeldsen SE, Makani H, Grossman E, et al (2011) Antihypertensive drugs and risk of cancer: network meta-analyses and trial sequential analyses of 324,168 participants from randomised trials. Lancet Oncol 12: 65–82. 21123111 10.1016/S1470-2045(10)70260-6

[3] U.S.Food and Drug Administration (2011) FDA Drug Safety Communication: No increase in risk of cancer with certain blood pressure drugs–Angiotensin Receptor Blockers (ARBs). U S Food and Drug Administration

[4] Pasternak B, Svanstrom H, Callreus T, Melbye M, Hviid A (2011) Use of angiotensin receptor blockers and the risk of cancer. Circulation 123: 1729–1736. 21482967 10.1161/CIRCULATIONAHA.110.007336

[5] Huang CC, Chan WL, Chen YC, Chen TJ, Lin SJ, et al (2011) Angiotensin II receptor blockers and risk of cancer in patients with systemic hypertension. Am J Cardiol 107: 1028–1033. 21256465 10.1016/j.amjcard.2010.11.026

[6] Hallas J, Depont CR, Andersen M, Friis S, Bjerrum L (2012) Long-term use of drugs affecting the renin-angiotensin system and the risk of cancer. A population-based case-control study. Br J Clin Pharmacol 10.1111/j.1365-2125.2012.04170.x PMC3394143 22243442

[7] Bhaskaran K, Douglas I, Evans S, van ST, Smeeth L (2012) Angiotensin receptor blockers and risk of cancer: cohort study among people receiving antihypertensive drugs in UK General Practice Research Database. BMJ 344: e2697. 22531797 10.1136/bmj.e2697 PMC3339864

[8] Walley T, Mantgani A (1997) The UK General Practice Research Database. Lancet 350: 1097–1099. 10213569 10.1016/S0140-6736(97)04248-7

[9] Garcia Rodriguez LA, Perez GS (1998) Use of the UK General Practice Research Database for pharmacoepidemiology. Br J Clin Pharmacol 45: 419–425. 9643612 10.1046/j.1365-2125.1998.00701.x PMC1873548

[10] Jick H, Jick SS, Derby LE (1991) Validation of information recorded on general practitioner based computerised data resource in the United Kingdom. BMJ 302: 766–768. 2021768 10.1136/bmj.302.6779.766 PMC1669537

[11] Lawrenson R, Williams T, Farmer R (1999) Clinical information for research; the use of general practice databases. J Public Health Med 21: 299–304. 10528957 10.1093/pubmed/21.3.299

[12] Lawrenson R, Todd JC, Leydon GM, Williams TJ, Farmer RD (2000) Validation of the diagnosis of venous thromboembolism in general practice database studies. Br J Clin Pharmacol 49: 591–596. 10848723 10.1046/j.1365-2125.2000.00199.x PMC2015047

[13] Jick SS, Kaye JA, Vasilakis-Scaramozza C, Garcia Rodriguez LA, Ruigomez A, et al (2003) Validity of the general practice research database. Pharmacotherapy 23: 686–689. 12741446 10.1592/phco.23.5.686.32205

[14] Jick H, Jick S, Derby LE, Vasilakis C, Myers MW, et al (1997) Calcium-channel blockers and risk of cancer. Lancet 349: 525–528. 9048789 10.1016/S0140-6736(97)80084-0

[15] Garcia-Rodriguez LA, Huerta-Alvarez C (2001) Reduced risk of colorectal cancer among long-term users of aspirin and nonaspirin nonsteroidal antiinflammatory drugs. Epidemiology 12: 88–93. 11138826 10.1097/00001648-200101000-00015

[16] Hall GC, Roberts CM, Boulis M, Mo J, MacRae KD (2005) Diabetes and the risk of lung cancer. Diabetes Care 28: 590–594. 15735193 10.2337/diacare.28.3.590

[17] Gonzalez-Perez A, Garcia Rodriguez LA (2005) Prostate cancer risk among men with diabetes mellitus (Spain). Cancer Causes Control 16: 1055–1058. 16184470 10.1007/s10552-005-4705-5

[18] van Staa TP, Patel D, Gallagher AM, de Bruin ML (2012) Glucose-lowering agents and the patterns of risk for cancer: a study with the General Practice Research Database and secondary care data. Diabetologia 55: 654–665. 22127412 10.1007/s00125-011-2390-3

[19] Friis S, Sorensen HT, Mellemkjaer L, McLaughlin JK, Nielsen GL, et al (2001) Angiotensin-converting enzyme inhibitors and the risk of cancer: a population-based cohort study in Denmark. Cancer 92: 2462–2470. 11745304 10.1002/1097-0142(20011101)92:9<2462::aid-cncr1596>3.0.co;2-l

[20] Tenenbaum A, Motro M, Jonas M, Fisman EZ, Grossman E, et al (2001) Is diuretic therapy associated with an increased risk of colon cancer? Am J Med 110: 143–145. 11165556 10.1016/s0002-9343(00)00674-4

[21] Assimes TL, Suissa S (2011) Immortal person time bias in pharmacoepidemiological studies of antihypertensive drugs. Am J Cardiol 108: 902–903. 10.1016/j.amjcard.2011.06.031 21884882

[22] Yoon C, Yang HS, Jeon I, Chang Y, Park SM (2011) Use of angiotensin-converting-enzyme inhibitors or angiotensin-receptor blockers and cancer risk: a meta-analysis of observational studies. CMAJ 183: E1073–E1084. 21876027 10.1503/cmaj.101497 PMC3185099

[23] Michels KB, Rosner BA, Walker AM, Stampfer MJ, Manson JE, et al (1998) Calcium channel blockers, cancer incidence, and cancer mortality in a cohort of U.S. women: the nurses' health study. Cancer 83: 2003–2007. 9806660

[24] Assimes TL, Elstein E, Langleben A, Suissa S (2008) Long-term use of antihypertensive drugs and risk of cancer. Pharmacoepidemiol Drug Saf 17: 1039–1049. 18780400 10.1002/pds.1656

[25] Assimes TL, Suissa S (2009) Age at incident treatment of hypertension and risk of cancer: a population study. Cancer Causes Control 20: 1811–1820. 19533392 10.1007/s10552-009-9374-3

