# Supplementary material for: Long-Term Use of Angiotensin Receptor Blockers and the Risk of Cancer
Source: PLoS One. 2012 Dec 12;7(12):e50893. doi: 10.1371/journal.pone.0050893 (PMC3521027; doi:10.1371/journal.pone.0050893)
Supplement: Table S1 — Characteristics of antihypertensive exposure groups among controls for lung cancer at index date. (DOC) [file pone.0050893.s001.doc]

| **Table S1** | | | | | |
| --- | --- | --- | --- | --- | --- |
| **Characteristics of antihypertensive exposure groups among controls for lung cancer at index date** | | | | | |
|  | **Diuretics and/or beta-blockers** | **ARBs** | **ACEIs** | **CCBs** | **Other AHDs** |
|  | **(n=24,426)** | **(n=14,487)** | **(n=39,668)** | **(n=21,189)** | **(n=2554)** |
| Excessive alcohol use, n (%) | 1465 (6.0) | 1214 (8.4) | 3601 (9.1) | 1546 (7.3) | 150 (5.9) |
| Body mass index, n (%) |  |  |  |  |  |
| < 18.5 kg/m^2^ | 306 (1.3) | 83 (0.6) | 374 (0.9) | 290 (1.4) | 25 (1.0) |
| 18.5-25 kg/m^2^ | 4990 (20.4) | 2253 (15.6) | 7141 (18.0) | 4398 (20.8) | 527 (20.6) |
| 25-30 | 6222 (25.5) | 3830 (26.4) | 10,549 (26.6) | 5561 (26.2) | 621 (24.3) |
| ≥ 30 | 3409 (14.0) | 3091 (21.3) | 7260 (18.3) | 2902 (13.7) | 425 (16.6) |
| Unknown | 9499 (38.9) | 5230 (36.1) | 14,344 (36.2) | 8038 (37.9) | 956 (37.4) |
| Smoking status, n (%) |  |  |  |  |  |
| Never | 12,160 (49.8) | 6523 (45.0) | 16,351 (41.2) | 9456 (44.6) | 1243 (48.7) |
| Ever | 10,224 (41.9) | 7650 (52.8) | 21,806 (55.0) | 10,425 (49.2) | 1168 (45.7) |
| Unknown | 2042 (8.4) | 314 (2.2) | 1511 (3.8) | 1308 (6.2) | 143 (5.6) |
| Previous cancer*, n (%) | 2175 (8.9) | 1562 (10.8) | 3799 (9.6) | 1959 (9.3) | 228 (8.9) |
| Diabetes, n (%) | 1592 (6.5) | 3386 (23.4) | 9564 (24.1) | 1846 (8.7) | 168 (6.6) |
| Aspirin, n (%)^a^ | 9050 (37.1) | 8280 (57.2) | 23,001 (58.0) | 10,803 (51.0) | 953 (37.3) |
| Statins, n (%) | 6097 (25.0) | 8212 (56.7) | 20,982 (52.9) | 7605 (35.9) | 683 (26.7) |
| NSAIDs, n (%) | 13,018 (53.3) | 8454 (58.4) | 21,276 (53.6) | 11,339 (53.5) | 1583 (62.0) |

Abbreviations: ARB, angiotensin receptor blocker; ACEI, angiotensin-converting enzyme inhibitor; CCB, calcium channel blocker; AHD, antihypertensive.

*Cancers other than non-melanoma skin cancer.
