# Supplementary material for: Long-Term Use of Angiotensin Receptor Blockers and the Risk of Cancer
Source: PLoS One. 2012 Dec 12;7(12):e50893. doi: 10.1371/journal.pone.0050893 (PMC3521027; doi:10.1371/journal.pone.0050893)
Supplement: Table S2 — Characteristics of antihypertensive exposure groups among controls for colorectal cancer at index date. (DOC) [file pone.0050893.s002.doc]

| **Table S2** | | | | | |
| --- | --- | --- | --- | --- | --- |
| **Characteristics of antihypertensive exposure groups among controls for colorectal cancer at index date** | | | | | |
|  | **Diuretics and/or beta-blockers** | **ARBs** | **ACEIs** | **CCBs** | **Other AHDs** |
|  | **(n=18,730)** | **(n=11,148)** | **(n=30,466)** | **(n=16,382)** | **(n=2026)** |
| Excessive alcohol use, n (%) | 1035 (5.5) | 948 (8.5) | 2621 (8.6) | 1113 (6.8) | 106 (5.2) |
| Body mass index, n (%) |  |  |  |  |  |
| < 18.5 kg/m^2^ | 254 (1.4) | 78 (0.7) | 306 (1.0) | 242 (1.5) | 23 (1.1) |
| 18.5-25 kg/m^2^ | 3911 (20.9) | 1844 (16.5) | 5510 (18.1) | 3525 (21.5) | 437 (21.6) |
| 25-30 | 4548 (24.3) | 2940 (26.4) | 7922 (26.0) | 4139 (25.3) | 487 (24.0) |
| ≥ 30 | 2667 (14.2) | 2289 (20.5) | 5522 (18.1) | 2178 (13.3) | 302 (14.9) |
| Unknown | 7350 (39.2) | 3997 (35.9) | 11,206 (36.8) | 6298 (38.4) | 777 (38.4) |
| Smoking status, n (%) |  |  |  |  |  |
| Never | 9466 (50.5) | 5152 (46.2) | 13,083 (42.9) | 7548 (46.1) | 1013 (50.0) |
| Ever | 7598 (40.6) | 5744 (51.5) | 16,094 (52.8) | 7759 (47.4) | 866 (42.7) |
| Unknown | 1666 (8.9) | 252 (2.3) | 1289 (4.2) | 1075 (6.6) | 147 (7.3) |
| Previous cancer*, n (%) | 1660 (8.9) | 1152 (10.3) | 2815 (9.2) | 1524 (9.3) | 205 (10.1) |
| Diabetes, n (%) | 1179 (6.3) | 2544 (22.8) | 7216 (23.7) | 1491 (9.1) | 152 (7.5) |
| Aspirin, n (%)^a^ | 6908 (36.9) | 6319 (56.7) | 17,510 (57.5) | 8329 (50.8) | 799 (39.4) |
| Statins, n (%) | 4316 (23.0) | 6226 (55.9) | 15,733 (51.6) | 5734 (35.0) | 539 (26.6) |
| NSAIDs, n (%) | 10,122 (54.0) | 6615 (59.3) | 16,252 (53.3) | 8819 (53.8) | 1181 (58.3) |
| Cholecystectomy, n (%) | 902 (4.8) | 676 (6.1) | 1442 (4.7) | 823 (5.0) | 134 (6.6) |
| Inflammatory bowel disease, n (%) | 246 (1.3) | 164 (1.5) | 426 (1.4) | 200 (1.2) | 32 (1.6) |
| History of polyps, n (%) | 284 (1.5) | 262 (2.4) | 594 (2.0) | 271 (1.7) | 36 (1.8) |

Abbreviations: ARB, angiotensin receptor blocker; ACEI, angiotensin-converting enzyme inhibitor; CCB, calcium channel blocker; AHD, antihypertensive.

* Cancers other than non-melanoma skin cancer.
