# Supplementary material for: Long-Term Use of Angiotensin Receptor Blockers and the Risk of Cancer
Source: PLoS One. 2012 Dec 12;7(12):e50893. doi: 10.1371/journal.pone.0050893 (PMC3521027; doi:10.1371/journal.pone.0050893)
Supplement: Table S3 — Characteristics of antihypertensive exposure groups among controls for prostate cancer at index date. (DOC) [file pone.0050893.s003.doc]

| **Table S3** | | | | | |
| --- | --- | --- | --- | --- | --- |
| **Characteristics of antihypertensive exposure groups among controls for prostate cancer at index date** | | | | | |
|  | **Diuretics and/or beta-blockers** | **ARBs** | **ACEIs** | **CCBs** | **Other AHDs** |
|  | **(n=24,324)** | **(n=15,182)** | **(n=50,211)** | **(n=24,793)** | **(n=1562)** |
| Excessive alcohol use, n (%) | 1870 (7.7) | 1730 (11.4) | 5348 (10.7) | 2210 (8.9) | 120 (7.7) |
| Body mass index, n (%) |  |  |  |  |  |
| < 18.5 kg/m^2^ | 175 (0.7) | 42 (0.3) | 270 (0.5) | 184 (0.7) | 10 (0.6) |
| 18.5-25 kg/m^2^ | 4899 (20.1) | 2177 (14.3) | 8936 (17.8) | 5146 (20.8) | 300 (19.2) |
| 25-30 | 6739 (27.7) | 4531 (29.8) | 14,584 (29.1) | 7198 (29.0) | 426 (27.3) |
| ≥ 30 | 3063 (12.6) | 3036 (20.0) | 8377 (16.7) | 2921 (11.8) | 196 (12.6) |
| Unknown | 9448 (38.8) | 5396 (35.5) | 18,044 (35.9) | 9344 (37.7) | 630 (40.3) |
| Smoking status, n (%) |  |  |  |  |  |
| Never | 9954 (40.9) | 5142 (33.9) | 16,716 (33.3) | 9269 (37.4) | 623 (39.9) |
| Ever | 12,340 (50.7) | 9684 (63.8) | 31,581 (62.9) | 13,929 (56.2) | 814 (52.1) |
| Unknown | 2030 (8.4) | 356 (2.3) | 1914 (3.8) | 1595 (6.4) | 125 (8.0) |
| Previous cancer*, n (%) | 2268 (9.3) | 1564 (10.3) | 4830 (9.6) | 2421 (9.8) | 169 (10.8) |
| Diabetes, n (%) | 1854 (7.6) | 3859 (25.4) | 13,042 (26.0) | 2418 (9.8) | 173 (11.1) |
| Aspirin, n (%)^a^ | 10,987 (45.2) | 9338 (61.5) | 31,556 (62.9) | 13,856 (55.9) | 921 (59.0) |
| Statins, n (%) | 6899 (28.4) | 9191 (60.5) | 28,187 (56.1) | 9481 (38.2) | 546 (35.0) |
| NSAIDs, n (%) | 11,695 (48.1) | 8356 (55.0) | 25,363 (50.5) | 12,532 (50.6) | 785 (50.3) |
| Benign prostatic hyperplasia, n (%) | 1739 (7.2) | 1339 (8.8) | 3792 (7.6) | 1907 (7.7) | 133 (8.5) |
| 5-alpha reductase inhibitors, n (%) | 1045 (4.3) | 992 (6.5) | 2697 (5.4) | 1379 (5.6) | 103 (6.6) |
| Number of PSA tests, mean (SD) | 0.32 (1.1) | 0.77 (1.8) | 0.51 (1.4) | 0.41 (1.3) | 0.34 (1.3) |

Abbreviations: ARB, angiotensin receptor blocker; ACEI, angiotensin-converting enzyme inhibitor; CCB, calcium channel blocker; AHD, antihypertensive.

*Cancers other than non-melanoma skin cancer.
