# Supplementary material for: Long-Term Use of Angiotensin Receptor Blockers and the Risk of Cancer
Source: PLoS One. 2012 Dec 12;7(12):e50893. doi: 10.1371/journal.pone.0050893 (PMC3521027; doi:10.1371/journal.pone.0050893)
Supplement: Table S4 — Characteristics of antihypertensive exposure groups among controls for breast cancer at index date. (DOC) [file pone.0050893.s004.doc]

| **Table S4** | | | | | |
| --- | --- | --- | --- | --- | --- |
| **Characteristics of antihypertensive exposure groups among controls for breast cancer at index date** | | | | | |
|  | **Diuretics and/or beta-blockers** | **ARBs** | **ACEIs** | **CCBs** | **Other AHDs** |
|  | **(n=34,243)** | **(n=16,000)** | **(n=35,521)** | **(n=21,160)** | **(n=6095)** |
| Excessive alcohol use, n (%) | 1308 (3.8) | 900 (5.6) | 1942 (5.5) | 965 (4.6) | 284 (4.7) |
| Body mass index, n (%) |  |  |  |  |  |
| < 18.5 kg/m^2^ | 469 (1.4) | 178 (1.1) | 502 (1.4) | 425 (2.0) | 76 (1.3) |
| 18.5-25 kg/m^2^ | 6828 (19.9) | 2613 (16.3) | 6628 (18.7) | 4629 (21.9) | 1264 (20.7) |
| 25-30 | 7336 (21.4) | 3577 (22.4) | 7439 (20.9) | 4609 (21.8) | 1295 (21.3) |
| ≥ 30 | 6283 (18.4) | 3911 (24.4) | 7956 (22.4) | 3431 (16.2) | 1100 (18.1) |
| Unknown | 13,327 (38.9) | 5721 (35.8) | 12,996 (36.6) | 8066 (38.1) | 2360 (38.7) |
| Smoking status, n (%) |  |  |  |  |  |
| Never | 19,755 (57.7) | 9138 (57.1) | 19,454 (54.8) | 11,829 (55.9) | 3159 (51.8) |
| Ever | 11,407 (33.3) | 6458 (40.4) | 14,383 (40.5) | 7853 (37.1) | 2583 (42.4) |
| Unknown | 3081 (9.0) | 404 (2.5) | 1684 (4.7) | 1478 (7.0) | 353 (5.8) |
| Previous cancer*, n (%) | 2545 (7.4) | 1527 (9.5) | 2835 (8.0) | 1677 (7.9) | 520 (8.5) |
| Diabetes, n (%) | 1569 (4.6) | 3120 (19.5) | 7168 (20.2) | 1589 (7.5) | 263 (4.3) |
| Aspirin, n (%)^a^ | 8306 (24.3) | 7598 (47.5) | 16,290 (45.9) | 9026 (42.7) | 1233 (20.2) |
| Statins, n (%) | 5628 (16.4) | 7858 (49.1) | 15,280 (43.0) | 6409 (30.3) | 1023 (16.8) |
| NSAIDs, n (%) | 19,533 (57.0) | 10,103 (63.1) | 20,622 (58.1) | 12,449 (58.8) | 4085 (67.0) |
| Oophorectomy, n (%) | 1205 (3.5) | 551 (3.4) | 1162 (3.3) | 692 (3.3) | 235 (3.9) |
| Hormone replacement therapy, n (%) | 10,147 (29.6) | 4624 (28.9) | 8945 (25.2) | 5059 (23.9) | 3510 (57.6) |
| Oral contraceptives, n (%) | 2932 (8.6) | 778 (4.9) | 1702 (4.8) | 915 (4.3) | 752 (12.3) |

Abbreviations: ARB, angiotensin receptor blocker; ACEI, angiotensin-converting enzyme inhibitor; CCB, calcium channel blocker; AHD, antihypertensive.

*Cancers other than non-melanoma skin cancer.
