# Supplementary material for: Long-Term Use of Angiotensin Receptor Blockers and the Risk of Cancer
Source: PLoS One. 2012 Dec 12;7(12):e50893. doi: 10.1371/journal.pone.0050893 (PMC3521027; doi:10.1371/journal.pone.0050893)
Supplement: Table S6 — Crude and adjusted rate ratios of cancer associated with antihypertensive agents relative to diuretic or beta-blocker use, stratified by new/prevalent user status. (DOC) [file pone.0050893.s006.doc]

| **Table S6** | | | |
| --- | --- | --- | --- |
| **Crude and adjusted rate ratios of cancer associated with antihypertensive agents relative to diuretic or beta-blocker use, stratified by new/prevalent user status** | | | |
|  | **Cases/Controls** | **Crude RR** | **Adjusted RR (95% CI)*** |
| **New-user cohort** | **18,856/188,338** |  |  |
| Diuretics/beta-blockers, n (%) | 5781/57,345 | 1.00 | 1.00 (Reference) |
| ARBs, n (%) | 2418/24,302 | 0.99 | 1.00 (0.95, 1.05) |
| ARBs with ACEIs, n (%)^‡^ | 115/1124 | 1.01 | 1.03 (0.84, 1.25) |
| ARBs without ACEIs, n (%) | 2303/23,178 | 0.99 | 1.00 (0.94, 1.05) |
| ACEIs, n (%) | 6776/68,194 | 0.99 | 0.99 (0.95, 1.03) |
| CCBs, n (%) | 3377/33,318 | 1.01 | 0.99 (0.95, 1.04) |
| Other antihypertensives, n (%) | 504/5179 | 0.96 | 0.94 (0.85, 1.04) |
|  |  |  |  |
| **Prevalent-user cohort** | **22,203/221,829** |  |  |
| Diuretics/beta-blockers, n (%) | 4310/44,378 | 1.00 | 1.00 (Reference) |
| ARBs, n (%) | 3165/32,515 | 1.00 | 1.00 (0.95, 1.06) |
| ARBs with ACEIs, n (%)^‡^ | 286/2753 | 1.08 | 1.07 (0.94, 1.21) |
| ARBs without ACEIs, n (%) | 2879/29,762 | 1.00 | 1.00 (0.95, 1.06) |
| ACEIs, n (%) | 9259/92,202 | 1.03 | 1.02 (0.98, 1.06) |
| CCBs, n (%) | 5245/50,655 | 1.07 | 1.05 (1.01, 1.10) |
| Other antihypertensives, n (%) | 224/2079 | 1.11 | 1.10 (0.96, 1.27) |

Abbreviations: RR, rate ratio; CI, confidence interval; ARB, angiotensin receptor blocker; ACEI, angiotensin-converting enzyme inhibitor; CCB, calcium channel blocker.

* Adjusted for excessive alcohol use, body mass index, smoking, diabetes, previous cancer, and ever of aspirin, statins, and NSAIDs.

^‡^ Defined as receiving prescriptions for both agents on the same day on at least one occasion.
