# Supplementary material for: Long-Term Use of Angiotensin Receptor Blockers and the Risk of Cancer
Source: PLoS One. 2012 Dec 12;7(12):e50893. doi: 10.1371/journal.pone.0050893 (PMC3521027; doi:10.1371/journal.pone.0050893)
Supplement: Table S7 — Crude and adjusted rate ratios of cancer associated with antihypertensive agents relative to diuretic or beta-blocker use, excluding any cancer before cohort entry. (DOC) [file pone.0050893.s007.doc]

| **Table S7** | | | |
| --- | --- | --- | --- |
| **Crude and adjusted rate ratios of cancer associated with antihypertensive agents relative to diuretic or beta-blocker use, excluding any cancer before cohort entry** | | | |
|  | **Cases/Controls** | **Crude RR** | **Adjusted RR (95% CI)*** |
| **cohort** | **38,066/385,836** |  |  |
| Diuretics/beta-blockers, n (%) | 9313/95,559 | 1.00 | 1.00 (Reference) |
| ARBs, n (%) | 5172/53,319 | 1.00 | 1.00 (0.96, 1.04) |
| ARBs with ACEIs, n (%)^‡^ | 2256/22,877 | 1.02 | 1.01 (0.96, 1.07) |
| ARBs without ACEIs, n (%) | 2916/30,442 | 0.99 | 0.99 (0.94, 1.04) |
| ACEIs, n (%) | 14,921/151,141 | 1.02 | 1.00 (0.97, 1.03) |
| CCBs, n (%) | 7989/78,978 | 1.04 | 1.03 (0.99, 1.06) |
| Other antihypertensives, n (%) | 671/6839 | 1.00 | 0.99 (0.91, 1.08) |

Abbreviations: RR, rate ratio; CI, confidence interval; ARB, angiotensin receptor blocker; ACEI, angiotensin-converting enzyme inhibitor; CCB, calcium channel blocker.

* Adjusted for excessive alcohol use, body mass index, smoking, diabetes, and ever of aspirin, statins, and NSAIDs.

^‡^ Defined as receiving prescriptions for both agents on the same day on at least one occasion.
