# Supplementary material for: Long-Term Use of Angiotensin Receptor Blockers and the Risk of Cancer
Source: PLoS One. 2012 Dec 12;7(12):e50893. doi: 10.1371/journal.pone.0050893 (PMC3521027; doi:10.1371/journal.pone.0050893)
Supplement: Table S8 — Crude and adjusted rate ratios of cancer associated with antihypertensive agents relative to diuretic and/or beta-blocker use, (non-mutually exclusive exposure groups). (DOC) [file pone.0050893.s008.doc]

| **Table S8** | | | |
| --- | --- | --- | --- |
| **Crude and adjusted rate ratios of cancer associated with antihypertensive agents (non-mutually exclusive exposure groups)** | | | |
|  | **Cases/Controls*** | **Crude RR** | **Adjusted RR (95% CI)**^†^ |
| Diuretics and/or beta-blockers | 34,808/347,776 | 1.00 | 1.00 (0.97-1.03) |
| ARBs | 5583/56,817 | 0.98 | 0.99 (0.96-1.02) |
| ACEIs | 19,910/199,737 | 0.99 | 0.99 (0.97-1.01) |
| CCBs | 20,285/198,892 | 1.04 | 1.04 (1.01-1.06) |
| Other antihypertensives | 1654/16,844 | 0.98 | 0.98 (0.93-1.03) |

Abbreviations: RR, rate ratio; CI, confidence interval; ARB, angiotensin receptor blocker; ACEI, angiotensin-converting enzyme inhibitor; CCB, calcium channel blocker; DDD, defined daily doses.

^†^ Adjusted for excessive alcohol use, body mass index, smoking, diabetes, previous cancer, and ever of aspirin, statins, NSAIDs, hypertension, congestive heart failure, and coronary heart disease.
